# The effect of work setting and demographic factors on caring behaviour among nurses in the public hospitals and public health services, Sabah, Malaysia

**DOI:** 10.1186/s12912-023-01359-w

**Published:** 2023-06-08

**Authors:** Norkiah Arsat, Nik Amin Sahid Nik Lah, Deena Clare Thomas, Sui Fun Soong, Li Tsu Chong, Waidah Sawatan, Norsimah Dasan, Walton Wider

**Affiliations:** 1 Department of Nursing, Faculty of Medicine and Health Science, Universiti Malaysia Sabah, Kota Kinabalu, Malaysia; 2 Department of Surgery, Faculty of Medicine and Health Science, Universiti Malaysia Sabah, Kota Kinabalu, Malaysia; 3 Nursing Department, Cyberjaya College, Kota Kinabalu, Malaysia; 4 Faculty of Psychology and Education, Universiti Malaysia Sabah, Kota Kinabalu, Malaysia; 5 Faculty of Business and Communications, INTI International University, Nilai, Negeri Sembilan Malaysia

**Keywords:** Caring behavior, Nurse, Public hospitals, Public health services, Malaysia

## Abstract

**Background:**

The nursing profession's uniqueness contributes to the development of knowledge, experience, age, education, economy, and position, as well as a unique gender role. Thus, the development and advancement of demographic characteristics of nurses while in this profession influences their caring behavior.

**Objective:**

The purpose of this study was to determine the effect of work setting and demographic factors on nurses' caring behaviour, as well as the differences in nurses' caring behaviour based on demographic factors between nurses in public hospitals and nurses in public health services in Sabah, Malaysia.

**Methods:**

This research is a cross-sectional study using the survey method. Data were collected from 3,532 nurses (88.3% response rate) in public hospitals and public health services within Sabah, Malaysia. Data were analysed using two-way ANOVA.

**Results:**

The two-way ANOVA test revealed no significant impact of the work setting on CB, nor was there a notable interaction between the work setting and demographic factors influencing nurses’ CB. However, demographic factors such as gender, age, education, economic status, position, and experience had a significant effect on CB.

**Conclusion:**

The present research has provided convergent evidence on the effect of demographic factors on nurses caring behavior and the differences in caring behavior based on demographic factors among nurses in public hospitals and public health services in Sabah, Malaysia.

## Introduction

Caring Behavior (CB) is a core value in the Ministry of Health Malaysia (MOH), integral to realizing its vision and mission of promoting and facilitating the use of health care services to achieve optimal health and a high-quality health system [1]. To reach these goals, the MOH has implemented several efforts to improve caring services. These include frequent caring service training for senior staff and newcomers to the health services department and encouraging all staff to display CB while serving patients or clients. Despite these initiatives, there is dissatisfaction with the health care services provided. The MOH receives approximately 7,000 complaints annually, ranging from issues with services and facilities to poor communication skills among doctors and nurses, long waiting times, and inadequate equipment [2].

Nurses, as the backbone of every health care organization, have a vital role in achieving national health service objectives. Given that caring is the essence of nursing [3], nurses are more closely associated with CB than other health care workers, thereby significantly influencing patient satisfaction [4, 5]. They represent the largest workforce in Public Hospital (PH) and Public Health Service (PHS) and provide round-the-clock patient care in both settings.

CB is frequently linked to nurses in PH due to their 24-hour shifts, whereas PHS nurses only interact with clients for 9 hours (office hours). This variance in interaction duration might influence nurses' CB performance in different work settings. Moreover, PH nurses attend to critically ill and chronic patients, while PHS nurses provide community health care services, resulting in distinct differences in workload, workplace environment, and patient or client characteristics. Previous studies have determined that factors such as workload, job satisfaction, workplace conditions, educational background, and patient characteristics affect nurses’ CB, with workload and job satisfaction being primary influencers [6, 7]. These factors present considerable challenges for nurses, particularly regarding their CB.

### Public Hospital (PH) and Public Health Service (PHS) in Sabah, Malaysia

PH nurses are tasked with providing nursing care to injured, ill, and disabled patients. They not only treat individual patients but also manage multiple patients concurrently. Conversely, in PHS, nurses serve the larger community, promoting potential health problems, health, nutrition, safety, and hygiene, and facilitating community access to health services. This discrepancy in duties often leads to perceived unequal workloads, with PH nurses seemingly engaged more frequently with CB than PHS nurses. Increases in nurses' workloads and decreases in their commitment to organisational goals can affect CB, impacting patient satisfaction [8].

While PH nurses face high CB demands, PHS nurses encounter their own challenges. Previous research indicates that nurses in remote settings perceive primary health care as a social model incorporating both community and individual care [9]. However, due to resource scarcity in remote locations, they often struggle to consistently provide quality health care. The lack of physical resources, limited access to specialized health services, and time constraints further hinder nurses in rural areas from delivering better primary health care. Additionally, despite increasing health care needs, there has not been a proportionate rise in the number of registered nurses in primary health care services (MOH Malaysia, 2020) [10]. This imbalance correlates with a decline in care quality and patient safety, negatively impacting the health of professional groups. The onset of burnout syndrome in primary health care nurses contributes to deteriorating care quality and patient safety [11], potentially presenting additional challenges to the practice of CB in PHS nurses.

Prior research suggests that the work environment influences nurses' caring behavior [6], but it doesn't delineate the differential effects on PH and PHS nurses. In PH, nurses focus on treating the ill and restoring health balance within the hospital setting. They follow up with admitted patients or provide one-time care. Tasks can be delegated at shift's end through a process known as clinical handover, which ensures continuity of patient care. Meanwhile, PHS nurses practice person-centered care (PCC), providing health services to individuals, families, and small groups in a community context. They can perform health assessments, determine health care needs, and make referrals, with full accountability for client care in health clinics, homes, and schools [12-14].

Previous studies have shown that a positive work environment and lower workload foster caring behaviors among nurses [15]. Aspects such as work environment characteristics, job satisfaction, and workload influence unsatisfactory caring behavior levels. Therefore, varying roles and responsibilities across different work settings likely influence nurses' caring behaviors due to the scope of their work. This study aims to delineate these distinctions.

### Nurses caring behavior

The theory of human care suggests that relationship-focused caring is essential for healing practices as it holistically honors humans while fostering a healing environment. A caring-healing approach combined with the art of nursing is vital to ensuring the focus is on the quality of life, the inner healing experience, and caring practices that influence patient outcomes. This approach aligns with a human-caring value-guided ethic for professional practice and existing nursing theory, meeting public expectations [16]. Caring behavior (CB) forms the philosophical and ethical foundation of professional nursing, a major focal point of the discipline considered both an art and a science. This foundation integrates art, science, humanities, spirituality, and new dimensions of mind-body-spirit medicine [17]. However, the application of CB can vary. Nurses' behaviors, including the application of CB, can differ depending on their work environments and demographic factors [18]. After accounting for these factors, research found that nurses with 15 years of experience rated their CB lower than those with 30 years of experience [19]. Notably, nurses holding a Bachelor of Science in Nursing (BSc) displayed a lower CB than those with a high school diploma. Interestingly, female nurses exhibited a higher level of caring behavior compared to male nurses [20], although other studies reported no gender difference in CB [21]. Studies from diverse regions such as Indonesia (e.g., [22]) and eastern Ethiopia (e.g., [15]) have reported no significant relationship between nurses' characteristics, such as work experience, gender, educational level, and caring behavior.

In Malaysia, a study exploring the impact of the working environment on nurses' caring behavior revealed demographic differences among nurses in public hospitals and public health services [6]. In terms of roles, positions included staff nurses, community nurses, head nurses, nurse supervisors, chief nurse supervisors, and clinical nurse specialists. Research has suggested a positive association between staff resources and caring behavior, with an inverse correlation seen with nurse managers [15]. As a result, various roles, responsibilities, and demographic factors in different work settings are predicted to influence nurses' caring behavior. Additionally, Putra et al. [23]found that certain dimensions of job satisfaction, namely supervision, contingent rewards, co-workers, nature of work, and communication, influenced nurses' caring behavior. However, factors like salary, promotion, benefits, or operating procedures were not significant in a military hospital context. Similarly, Mutmainnah et al. [20] discovered links between organizational, psychological, and spiritual factors and a nurse's caring behavior at Jambi Teaching Hospital, with no relation to demographic factors.

The uniqueness of the nursing profession contributes to the development of knowledge and experience as demographic factors such as age, education, economy, and position increase. Additionally, a distinct gender role forms over the course of a nurse's career. Despite extensive studies on CB in other countries, research in Malaysia, especially in relation to demographic factors and work setting, remains limited [5, 6]. Given these varying findings and the recognized importance of CB in improving patient care and satisfaction [24], there is a clear need for more research into Sabahan nurses' caring behavior [6]. Therefore, this study aims to investigate the effect of work setting and demographic factors on nurses' caring behavior, as well as any differences in CB based on these factors, between nurses in public hospitals and public health services in Sabah, Malaysia.

## Methods

### Design

This research is a cross-sectional study using the survey method to examine the effect of work setting and demographic factor on nurse’ CB and to investigate the difference in CB based on demographic factors (gender, age, education, economic status, position, and experience) between nurses in PH and PHS.

### Participants

Sabah, located on the island of Borneo, is one of the 13 states of Malaysia. It's known for its rich biodiversity, diverse cultures, and extensive healthcare network, which is spread out over both urban and rural areas. It has a well-established system of public health services that extends beyond traditional hospital settings into health clinics, maternal and child health clinics, rural clinics, travel clinics, and 1 Malaysia Clinics. These clinics offer a variety of inpatient services and act as a vital part of the state’s healthcare system, providing essential care to Sabah's communities. In this study, a total of 3,532 nurses, representing a broad cross-section of the nursing profession, participated. The inclusion criteria for this study encompassed registered nurses from various grades and categories, as well as those in management and professional groups. This includes nurses who provide care not only in public hospitals but also across a spectrum of public health services. The specific process of selecting respondents for this study has been detailed in Arsat et al. [6], ensuring a comprehensive and diverse representation of nurses from the state of Sabah, Malaysia.

Overall, a total of 4000 questionnaires were distributed to the respondents. The response rate was *n* = 3867 (96.68%). However, during the process of data entering, two questionnaires were found not filled, three questionnaires were unusable due to missing data, and three questionnaires had similar responses presumably filled by the same respondent. Next, a straight line was identified in 327 responses in which respondents gave a similarly high response rate in the questionnaire, which was considered a biased response to the data [25]. This brought the total number of questionnaires that could be used to *n*=3532 (88.3%) which was considered a very high response rate.

### Measurement

The questionnaire consists of a demographic information (age, economic status, education level, position, and working experience) section and The 24-item CB Inventory (CBI-24) which is considered to be the third-generation instrument for the measurement of caring [26]. The current study adopts the CBI-24 by Wu et al. [27] to explore the perception of the frequency of CB as practiced by nurses. It is based upon a conceptual definition of nurse caring as an interactive and inter-subjective process that occurs during moments of shared vulnerability between nurses and patients [21]. This scale consists of four components, namely, "assurance of human presence" (8 items), which deals with patients' needs and security; "knowledge and skill" (5 items), related to nurses as skillful and educated persons; "respectful deference to the other" (6 items), dealing with how nurses show interest in the patients; and "positive connectedness" (5 items), which corresponds to the need for nurses to be ready to help patients [27]. For each item, respondents are requested to answer using a 6-point Likert scale (1 = never and 6 = Always). The CBI-24 demonstrated good internal consistency, Cronbach's α = .96 (Wu et al. [27]). The researchers translated the CBI-24 into the Malaysian language and requested help from bilingual experts (two Malaysian nursing experts who can read and write in Malay and English) to translate the translated instrument (Malay version) back into the English version using the back-translation technique.

### Reliability of the instrument

A pilot study was conducted to ensure the suitability of this instrument in the local context as this instrument is from abroad. Respondents were composed of various categories of nurses from PH and PHS who came to the College of Allied Health Sciences to attend the local preceptor course. A total of 120 questionnaires were distributed before the program started, only 101 questionnaires were returned, only 95 questionnaires were filled in the demographic data section, and 98 questionnaires were filled in the Nurse Behavior Scale section. The results of the reliability evaluation of the 24-Item CB Inventory (CBI-24) instrument, were found to be at the overall level of Cronbach's alpha reliability coefficient of .960. The results of the reliability assessment for each scale found different Cronbach's Alpha values. The highest Cronbach's Alpha coefficients, was the 'assurance" (Cronbach's alpha = .912), followed by the scale of "respectful" to patients scale (Cronbach's alpha = .887), the "knowledge and skills" scale (Cronbach's alpha = .870), and the "connectedness" scale (Cronbach's alpha = .821).

### Data collection

This study used multistage cluster sampling to collect data. At the first stage, multistage cluster sampling was used to choose hospitals and district health offices. Followed by selecting the larger hospitals that had many wards and units, and district health offices that had many health clinics, rural clinics, and other units. For PHS, seven district health offices out of 24 in the state were chosen involving 10 health clinics, nine maternal and child health clinics, 73 rural clinics, and three traveling or mobile clinics. As for PH, a total of 12 hospitals were chosen with a total of 244 wards and units out of 24 hospitals across the state. At the second stage, the sample was clustered according to ward or units in PH and health clinics, rural clinics, and other units in the PHS for distribution of questionnaires.

Before data collection, the researchers met with every hospital director, hospital matrons, area health officer, and district health matrons to discuss the administering of questionnaires. They proposed that the questionnaires be administered by the nursing sister (also known as ward sister or unit sister) or nurse-in-charge to avoid disruption to the nurses on duty. All personnel involved in the data collection procedure were briefed on how to administer the questionnaire on purpose, confidentiality, how to collect the data, how to respond to any respondents' inquiries and to inform the respondents that they had the right to decline to answer any question for any particular reason or withdraw from the study at any time. Completed questionnaires were kept in sealed envelopes or sealed paper boxes to ensure confidentiality and were not accessible to anyone.

To collect completed questionnaires, the researchers and research assistants re-visited each research site, though some officers, matrons, nursing sisters, and nurses were kind enough to volunteer to send the completed questionnaires by mail or through officially recognized individuals. Nevertheless, some challenges arose in the collection process. First, the geographical location of hospitals throughout the state is such that road access is difficult and takes time, especially for health clinics which are mostly located in remote areas. Also, some of the responsible person for administering the data collection unable to cooperate even though the researchers had explained the purpose of the study along with evidence of ethical considerations. Therefore, the study location was shifted to the nearest hospital or clinic that was willing to participate.

### Data analysis

Prior to undertaking the data analysis, we implemented measures to guarantee data integrity during the transition from physical questionnaires to our digital database. We utilized a double-entry method, in which two team members independently entered the same set of data. We then compared the datasets, referred back to the original questionnaires to resolve discrepancies, and cross-verified a random sample of entries for additional verification. The IBM Statistical Package for Social Sciences (SPSS) version 26.0 program was used for data analysis [28]. Descriptive analysis was performed to calculate means, standard deviations, and frequencies to represent demographic profiles such as gender, age, education level, economy, position, and experience. A two-way ANOVA was utilized to identify the effects of work setting and demographic factors on nurses' caring behavior and to analyze the differences in caring behavior based on demographic factors between public health services and public hospitals. The significance level was set at *p*<0.05 and *p*<0.001.

## Results

In this study, we analyzed the mean values obtained from survey responses across several demographic variables, such as age, education, economic status, job position, and years of experience. Regarding age, nurses in the 50-59 years group had the highest mean values in both Public Hospitals (PH) (5.4264) and Public Health Services (PHS) (5.3352), whereas those in the 20-29 years group had the lowest mean values in both PH (5.1351) and PHS (5.1452). For education, PH nurses with a PhD recorded the highest mean score (5.5417); no PhD nurses were in PHS. Nurses holding a certificate reported the lowest mean values in both PH (5.1440) and PHS (5.2354). Economically, nurses identifying as "Luxurious" had the lowest mean score in PH (4.6019), while those considering themselves "Above Average" had the highest mean scores in both PH (5.4894) and PHS (5.4413). In terms of position, the highest mean score in PH (5.5417) came from nurses in the Head Nursing Matron (NSpvr U41) role. In PHS, however, the highest mean score was among the Nursing Matron (NSpvr U42) group (5.7639). The lowest mean scores were from Assistant Nurses (AN U11) in PH (4.8778) and Assistant Nurses (AN U14) in PHS (4.8403). Regarding experience, nurses with 30-35 years of experience had the highest mean score in both PH (5.4528) and PHS (5.3716), while those with less than 5 years of experience had the lowest mean scores in both PH (5.1164) and PHS (5.1611).

**Fig. 1:**
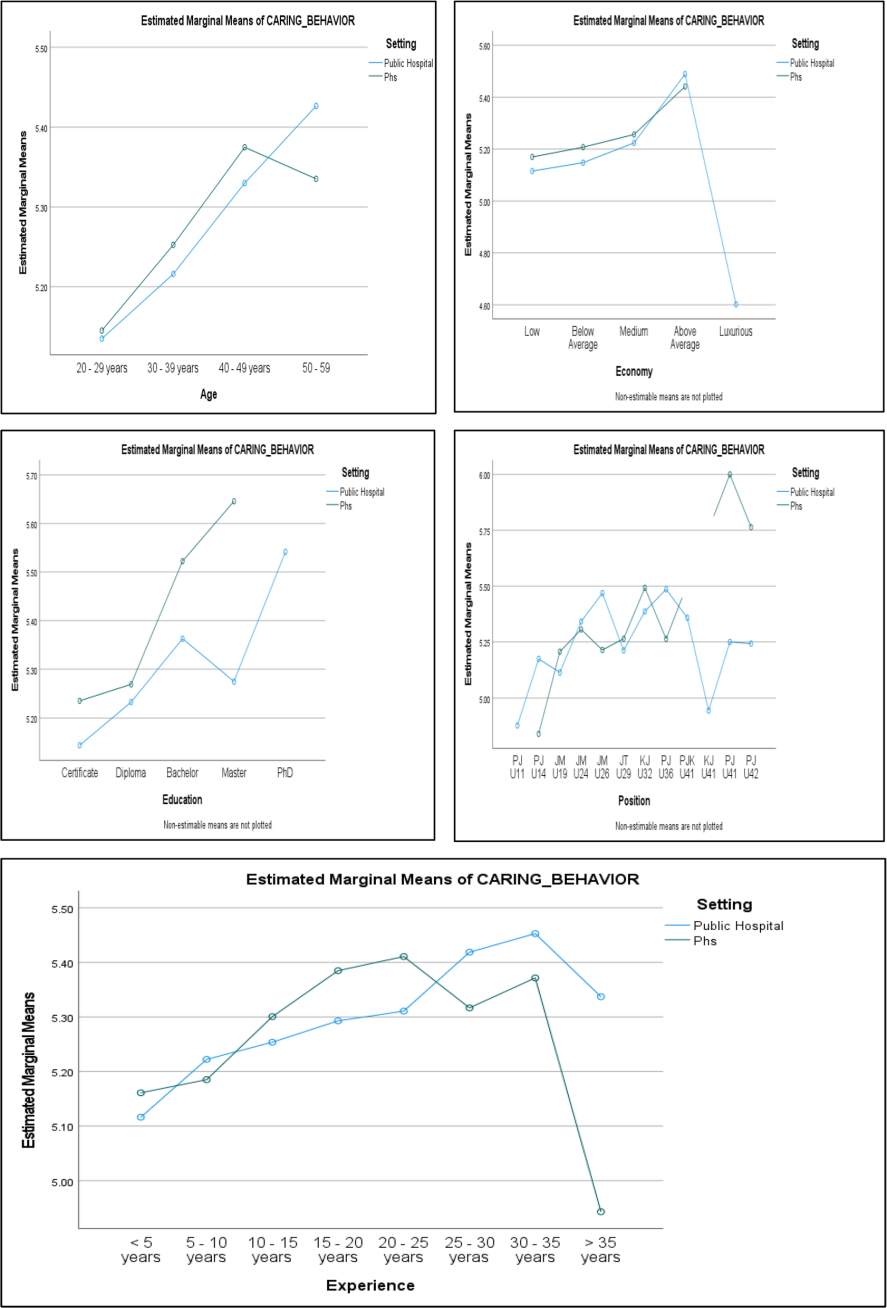
Estimated marginal means

The two-way ANOVA test results did not indicate a significant effect of the work setting on CB. Likewise, the interaction between the work setting and demographic factors did not show a substantial effect on nurses' CB. However, demographic factors (gender, age, education, economy, position, and experience) significantly influenced CB (see Tables 1 and 2). The estimated marginal mean results revealed a considerable difference in nurses' CB between PH and PHS, as illustrated in Figure 1.

**Table 1:** Demographic profile

**Variable**	**Setting**	**Mean**	**SD**	**N**
**Age**				
20 - 29 years	PH	5.1351	.63808	1102
	PHS	5.1452	.61189	293
	Total	5.1372	.63246	1395
30 - 39 years	PH	5.2161	.66004	736
	PHS	5.2525	.61826	478
	Total	5.2304	.64390	1214
40 - 49 years	PH	5.3299	.60532	381
	PHS	5.3748	.61022	201
	Total	5.3454	.60687	582
50 - 59	PH	5.4264	.60762	230
	PHS	5.3352	.56395	111
	Total	5.3967	.59447	341
Total	PH	5.2171	.64379	2449
	PHS	5.2547	.61426	1083
	Total	5.2286	.63503	3532
**Education**				
Certificate	PH	5.1440	.67955	606
	PHS	5.2354	.60647	687
	Total	5.1926	.64313	1293
Diploma	PH	5.2329	.63401	1724
	PHS	5.2695	.62923	368
	Total	5.2393	.63318	2092
Bachelor	PH	5.3628	.56244	113
	PHS	5.5224	.57024	26
	Total	5.3927	.56528	139
Master	PH	5.2750	.49965	5
	PHS	5.6458	.02946	2
	Total	5.3810	.44646	7
PhD	PH	5.5417	.	1
	Total	5.5417	.	1
Total	PH	5.2171	.64379	2449
	PHS	5.2547	.61426	1083
	Total	5.2286	.63503	3532
**Economy**				
Low	PH	5.1156	.67734	142
	PHS	5.1700	.62636	63
	Total	5.1323	.66104	205
Below Average	PH	5.1480	.65050	455
	PHS	5.2076	.61993	166
	Total	5.1639	.64252	621
Medium	PH	5.2241	.63153	1698
	PHS	5.2566	.61416	787
	Total	5.2344	.62614	2485
Above Average	PH	5.4894	.55533	145
	PHS	5.4413	.56121	66
	Total	5.4743	.55629	211
Luxurious	PH	4.6019	1.52953	9
	Total	4.6019	1.52953	9
Total	PH	5.2171	.64379	2449
	PHS	5.2553	.61420	1082
	Total	5.2288	.63503	3531
**Position**				
Assistants Nurse (AN U11)	PH	4.8778	.74660	15
	Total	4.8778	.74660	15
Assistants Nurse (AN U14)	PH	5.1745	.61260	16
	PHS	4.8403	.67773	6
	Total	5.0833	.63295	22
Community Nurse (CN U19)	PH	5.1140	.68520	506
	PHS	5.2068	.61805	566
	Total	5.1630	.65195	1072
Community Nurses (CN U24)	PH	5.3417	.58876	25
	PHS	5.3062	.57018	123
	Total	5.3122	.57148	148
Community Nurse (CN U26)	PH	5.4688	.52693	8
	PHS	5.2143	.57080	14
	Total	5.3068	.55673	22
Staff Nurses (SN U29)	PH	5.2124	.63031	1518
	PHS	5.2649	.62519	277
	Total	5.2205	.62963	1795
Nursing Sister (NSr U32)	PH	5.3871	.61407	328
	PHS	5.4925	.52780	78
	Total	5.4073	.59929	406
Nurse Supervisor (NSpvr U36)	PH	5.4861	.45170	15
	PHS	5.2639	.77323	15
	Total	5.3750	.63238	30
Head Nursing Matron (NSpvr U41)	PH	5.3583	.61633	5
	Total	5.3583	.61633	5
Nursing Sister (NSr U41)	PH	4.9444	.20972	3
	Total	4.9444	.20972	3
Nursing Matron (NSpvr U41)	PH	5.2500	.63738	4
	PHS	6.0000	.	1
	Total	5.4000	.64590	5
Nursing Matron (NSpvr U42)	PH	5.2431	.79862	6
	PHS	5.7639	.20554	3
	Total	5.4167	.69065	9
Total	PH	5.2171	.64379	2449
	PHS	5.2547	.61426	1083
**Experience**				
<5 years	PH	5.1164	.64388	991
	PHS	5.1611	.62181	238
	Total	5.1250	.63966	1229
5 - 10 years	PH	5.2223	.64257	530
	PHS	5.1851	.62394	285
	Total	5.2093	.63598	815
10 - 15 years	PH	5.2537	.63457	313
	PHS	5.3006	.61394	265
	Total	5.2752	.62509	578
15 - 20 years	PH	5.2929	.61863	199
	PHS	5.3849	.59968	114
	Total	5.3264	.61243	313
20 - 25 years	PH	5.3108	.62145	131
	PHS	5.4107	.54642	56
	Total	5.3407	.60027	187
25 - 30 years	PH	5.4187	.66592	188
	PHS	5.3167	.52275	65
	Total	5.3925	.63282	253
30 - 35 years	PH	5.4528	.53145	75
	PHS	5.3716	.58814	49
	Total	5.4207	.55362	124
>35 years	PH	5.3371	.50705	22
	PHS	4.9432	.78024	11
	Total	5.2058	.62811	33
Total	PH	5.2171	.64379	2449
	PHS	5.2547	.61426	1083
	Total	5.2286	.63503	3532

**Table 2:** Effect of work setting and nurses’ demography on caring behavior

**Source**	**df**	**Mean Square**	**F**	**Sig.**	***adj. R***^***2***^
**Variable 1**					
Age	3	7.285	18.426	<.001	
Work Setting	1	1.193	.000	.999	
Age * Setting	3	.366	.927	.427	
					.020
**Variable 2**					
Education	4	1.713	4.268	.002	
Work Setting	1	.575	1.433	.231	
Education * Setting	3	.262	.652	.582	
					.005
**Variable 3**					
Economy	4	4.068	10.222	<.001	
Work Setting	1	.176	.441	.506	
Economy * Setting	3	.135	.339	.797	
					.013
**Variable 4**					
Position	11	1.990	5.006	<.001	
Work Setting	1	.191	.482	.488	
Position * Setting	8	.397	1.000	.434	
					.014
**Variable 5**					
Experience	7	3.101	7.852	<.001	
Work Setting	1	.442	1.119	.290	
Experience * Setting	7	.527	1.334	.229	
					.021

^*^means interaction

## Discussion

The purpose of this study was mainly to examine the effects of work setting and demographic factors on nurses’ CB. This study also considers the differences in CB based on demographic factors between nurses in PH and PHS. The results showed that there was no major effect of work setting on nurses’ CB. The effect of interaction between setting and demographic factors did not show a significant effect on nurses’ CB. The result shows that only the demographic factors (age, education, economy, position, and experience) had significant effects on nurses’ CB. Thus the alternative hypothesis for the effect of work setting and demographic factors on nurses’ CB is partially rejected. Contrary, other authors found that there was no significant relationship between nurses’ CB with demographic factors (age, gender, and marital status) [15, 22]. Nevertheless, there is a significant relationship between nurses’ CB and their experience and workload for all caring dimensions as reported by Shalaby et al. [7].

The current study found that there were significant differences in nurses’ caring behaviors based on demographic factors between nurses working in public hospitals and public health services. Although fewer studies are examining the differences in nurses CB based on demographic factors between settings, Zhang et al. [18] in their study on the relationship between ethical climates and nursing service behavior in public hospital and private hospitals has proven that there are differences in nurses’ behavior according to where they work. They found that the demographic factors (age, work experience, and education level) have significant differences between different work environments. They argue that the work environment climate factors greatly influence the behavior and practice of nurses which greatly influences patient satisfaction and also reflects the image of the health provider organization. For example, in PH, in addition to performing direct nursing care, nurses also perform indirect nursing care which contributes to the workload of nurses. Shalaby et al. [7], found that 80% of military hospital critical care unit nurses perceived factors of workload, job satisfaction, workplace conditions, and educational background, as well as patient characteristic traits, were highly influencing nurses’ CB and were a major challenge for nurses. Among these factors, workload and job satisfaction were placed by nurses as the first category factors that influence nurses’ CB. Their finding supported Oluma’s study in which they revealed that nurses who had personal satisfaction with their jobs had high CB [29].

The results of the two-way ANOVA showed that the age factor had a significant effect [F(3,3524) = 18.43, *p*<.05] on nurses’ CB. The sample aged 20-29 years showed a lower level of CB in PH compared to those in the PHS. The level of CB was found to increase when reaching the age of 40-49 in both settings. However, the nurses’ CB will decrease when it reaches the age of 50-59 years for samples in the PHS but continues to increase for samples in PH until they reach the age of 50-59 years. Zhang et al. [18] identified the group differences in in-role and extra-role service behavior showed that nurses who work in a PH have a high level of in-role and extra-role service behavior were aged above 40 years. This indicates that the above 40-year-old sample has high behavior in performing the required tasks or can fulfill the core duties and responsibilities in caring for patients. They also had a high level of action behavior in performing tasks that were not included in the actual task but were related to their position or role as a nurse that added value to the client and health care provider [30]. This may be attributed to nurse's maturity in the above 40-year-old and 50-59 years old age group that has more work experience that contributes to more skill full in delivering nursing care in tandem with nurses’ CB in the PH setting. While in the PHS nurses in the age category of 50-59 years are senior nurses who are usually among the nurse supervisors or head nurses who mostly perform supervisory duties on junior nurses and perform more administrative duties.

The results of the two-way ANOVA showed that the education factor had a significant effect [F(4,3523) = 4.26, *p*<.05] on nurses’ CB. The PH sample showed a lower level of CB compared to the PHS samples for all level of education. There was an increase in the level of CB started from certificate level of education until bachelor level of education, however, the level of CB showed a significant decrease at the level of masters and Ph.D. As Vujanić et al. [19] argue that this is because nurses with Bachelor of Nursing level of education are oriented towards organizational work, management, administrative work, and communication with other healthcare professionals. Therefore, nurses with a higher level of education have less time to interact directly with their patients. As for the level of CB in the sample of PH, showed a significant increase consistently along with the increase in the level of education.

The results of the two-way ANOVA showed that the economy factor had a significant effect [F(4,3531) = 10.22, *p*<.05] on nurses’ CB. The low to medium economic status of the PH sample showed a lower level of CB compared to the sample of PHS. While levels of CB in PHS and PH continue to rise, however, samples in PHS showed higher nurses CB at above-average economic compared to PH samples. Whereas only a sample in PH had a luxurious economic but showed a very significantly decreased level of CB. Putra et al. [23] found that salaries and rewards received by nurses were found to affect nurses’ CB. The results of their study lead to a negative direction that the greater the salary received by the nurses, the lower the CB of the nurses.

The results of the two-way ANOVA showed that the position factor had a significant effect [F(11,3532) = 5.00, *p*<.05] on nurses’ CB. The result indicates that the lowest level of CB among all nurses was ANU11 in PH. The ANU14 in the PHS showed the lowest level of CB compared to the PH. Both of these positions have limited qualifications in the field of nursing therefore they usually do indirect nursing care. As for the CNU19 in the PHS showed higher CB levels than in PH. This position has a specialized training certificate in the field of community health nursing. Therefore, their CB is higher in PHS because they are more proficient in their knowledge and skills in PHS. In contrast, CNU24 and U26 in PHS showed lower CB levels than in PH. In addition to having specialized knowledge and skills in the field of community nursing they are also given the responsibility of supervising CNU19, and help prepare the report in PHS. This to some extent attracts part of their time to be with clients. Unlike in PH, they are more involved with direct nursing care. SNU29 and NsrU32, showed a high level of CB in PHS compared to those who work in PH. The categories of these two positions are very important in both settings either with or without the required advanced diploma or post-basic courses. In PHS, their function is to carry out family health care services [31]. This suggests that nurses at PHS are more focused and in control of their clients’ health care and only refer to physicians if problems and risks are identified. While at PH, they conduct nursing care, and assist doctors in patient care. In addition, they also supervise AN, CN, and other subordinate staff. They also assist NsrU32 in ward administration and supervise trainees as well as perform other tasks based on instructions from superiors and other professionals in the patient care team. Therefore, this may cause them to be burdened with indirect nursing care that may affect their job satisfaction as a nurse which may influence CB [7, 29].

Meanwhile, NSpvrU36 in PHS showed lower CB levels than in PH. This is because this position category is senior and experienced nurses in the field of PHS. They are therefore involved in nursing management to ensure that PHS activities run smoothly. Whereas, in PH they together with junior nurses carry out nursing care on patients because of their extensive knowledge and experience in nursing care therefore they more often apply CB. Meanwhile, NSpvrU41 and NSpvrU42 in PHS showed higher CB than PH. This is because this position category, has their specific nursing qualifications and Bachelor of Nursing in addition to extensive experience in the field of PHS, therefore they also along with junior nurses handle health services on clients. While in PH they are given the task of nursing administration. Similarly, for nurses in the categories of NSpvr U41, NSrU41, SPvr U41, and NPvr U42 posts. Only in PH had Head of NSpvr U41, NSr U41, SPvr U41, and NPvr U42 samples. Therefore, no comparison can be made.

The results of the two-way ANOVA showed that the experience factor had a significant effect [F(7,3532) = 7.85, *p*<.05] on nurses’ CB. The samples in PHS with experience below 5 years showed lower CB levels compared to PH samples. The nurses CB in PH increased when they had served between 10 and 35 years. Although there is a slight decrease when work experience exceeds 35 years, nurses in PH remain higher level of CB compared to PHS. Zhang et al. [18] found that nurses in a PH with work experience of 11-15 years have a high level of in-role behavior and nurses with 16 to 20 years of experience have higher extra-role behavior. In-role performance refers to the behavior of individuals who perform tasks and responsibilities that have been set. While extra-role behavior refers to the performance of behavior beyond the expectations of an employee's role [32]. This suggests that the more experienced the nurses are, the higher their behavioral performance in caring for patients. Therefore, CB between 10 to 35 years of work experience is higher in PH. As Zhang et al. [18] found that, nurse service behavior will increase with increasing age (aged over 40 years), work experience (11-15 years and 16-20), have a bachelor's degree or master's degree or higher, and had a senior nursing professional title are those who work in PH. Meanwhile, nurses working in PHS showed a decline in CB levels starting after 25 years to more than 35 years. As explained earlier in PHS aged 50 to 59 years has a lower level of CB than the sample in the PH due to this age category is an experienced nurse who holds management and administrative positions and does less clinical work in the PHS. Therefore, CB levels will decrease when nurses have more than 25 to 35 years of work experience in PHS as most of them will be promoted and carry out nurse management roles and responsibilities.

Overall, the results of this study show that demographic factors have a significant effect on the CB of nurses. An alternative hypothesis is partially supported. An alternative hypothesis for differences in nurses’ CB based on demographics factors between nurses in PHS and PH is supported. The level of nurses CB in PHS is higher than nurses in the PH. This is contrary to the argument at the beginning that the CB of nurses in PH is higher than in PHS.

### Limitations

Studies in examining the effects of work setting and demographic factors on nurses caring behavior and to identify the differences in nurses’ CB based on demographic factors between PHS and PH are relatively lacking. Therefore, it is quite difficult to compare the results of this study with the finding of previous studies. Like other studies, this study also faces limitations. First, the questionnaires were distributed through various levels, which should be distributed through the top management in each PH and PHS. Then distributed to Nursing Matron or Nursing Sister for distribution to wards and units in PH and health clinics, and rural clinics in PHS before distributed to the respondents. This is for the reasons to avoid interference to the nurses who are on duty. Therefore, confidentiality is beyond the control of the researcher as a researchers have no opportunity to collect data face to face. It is recommended that future studies use observation methods on direct and indirect nursing care and caring behavior of nurses. Second, this study was a cross-sectional survey in which no explanation was given to explain the relationship between nurses’ CB and demographic factors for both settings. The generalization of the findings of this study is potentially limited, as this study was only conducted in Sabah, Malaysia. It is hoped that the results of this study can be extended by researchers who are interested in studying this matter in the future.

### Implications for nursing management

The findings of the present study play a crucial role in guiding future research and advancing nursing practices in Malaysia. The study found no significant relationship between the work setting and nurses' caring behaviors (CB), suggesting that other environmental or organizational influences may be investigated in future research. In contrast, demographic factors such as age, education, economic status, position, and experience had a significant impact on CB, indicating that interventions designed to improve CB should target these areas. In addition, this study revealed differences in CB regarding these demographic factors between nurses in public hospitals and public health services, highlighting the need to tailor interventions for nurses in various settings.

The findings revealed specific nuances in CB, providing nursing administrators and policymakers with crucial insights for designing targeted strategies to improve CB. The research also revealed areas of disagreement with previous studies, reflecting the complex web of factors influencing CB and pointing to areas requiring additional research. This knowledge of the factors influencing CB can be used to develop effective interventions for enhancing CB and patient satisfaction, as well as to meet the needs of the nursing staff. In summary, the study makes a substantial contribution to the growing body of literature on nurses' caring behaviors and provides invaluable insights for future research, policy formulation, and nursing practices in Malaysia.

## Conclusion

Nurses form the largest group of workers in PH and PHS. Overall, the CB of nurses in the PHS was higher than that of nurses in PH as nurses in the PHS had greater control over their duties and responsibilities as nurses. It is hoped that this study can attract the attention of local researchers to explore further related to the perspective of local nurses, especially to determine the relationship between nursing staff, resource adequacy, support, and CB. The results of this study can help to understand the caring behavior of nurses concerning their demographic characteristics to provide quality care with the best patient outcomes and provide satisfaction to health care recipients. This study has added to theoretical contributions in the academic and research fields as well as in practical implications in the field of nursing practice by addressing the effects of demographic factor on CB and the differences in CB based on demographic factors between nurses in PHS and PH. Through a literature search, this study is the first local study to evaluate the CB based on demographic factors among nurses in PHS and PH.

## Data Availability

The data that support the findings of this study are available from Ministry of Health Malaysia, but restrictions apply to the availability of these data, which were used under license for the current study, and so are not publicly available. Data are however available from the authors upon reasonable request and with permission of Ministry of Health Malaysia.

## Abbreviations

CB: Caring behavior
MREC: Medical Research and Ethics Committee
MOH: Ministry of Health Malaysia
NMRR: National Medical Research Register
PH: Public Hospital
PHS: Public Health Service
